# Reliability of Rapid Diagnostic Tests in the diagnosis of malaria amongst children in two communities in South West Nigeria

**DOI:** 10.1186/1475-2875-13-S1-P10

**Published:** 2014-09-22

**Authors:** Ibrahim S Bello, Jerome B Elusiyan, Babatunde I Awokola, Akinjide O Ogundokun, Bridget Omisore, Olanrewaju Oyegbade, Samuel Anu Olowookere, Emmanuel Akintunde Abioye-Kuteyi, Joseph O Elujoba

**Affiliations:** 1 Department of Family Medicine, Obafemi Awolowo University Teaching Hospital, Ile Ife, Osun State, Nigeria

## Background

Prompt treatment of malaria following adequate diagnosis helps to reduce morbidity and mortality in children. Lack of resources and adequate manpower in developing countries make malaria microscopy difficult. The Rapid Diagnostic Test kit (RDT) remains unpopular despite its availability and ease of use because of lack of local research on its effectiveness leading to over-prescription of Artemisinin Combination Therapy (ACT).

## Materials and methods

A total of 132 children age range 1-9 years were screened for malaria routinely with rapid diagnostic test kits (RDTs HRP 2) and malaria microscopy at the general outpatient department of OAU Teaching Hospital as well as at a comprehensive health centre at Imesi Ile, South West Nigeria while marking the 2013 World Malaria Day. Needle prick was used to collect blood sample for thick and thin smear. Giemsa stain was used to prepare the slide for microscopic examination.

## Results

A total of 132 children were recruited into the study. There were more females (59.1%) than males (40.9%). 35 (26.5%) children tested positive while 97 (73.5%) children tested negative for *Plasmodium falciparum*. In the Microscopy category, 35 (26.5%) children tested positive while 97 (73.5%) tested negative for malaria parasite. Out of the 35 children tested positive, RDT picked 33 as positive and 2 as negative, (sensitivity = 94.3%). While out of the 97 that tested negative for microscopy, RDT picked 94 as negative and 3 as positive (specificity = 96.9%). The positive predictive value and negative predictive values are 91.7% and 97.9% respectively.

## Conclusions

Rapid Diagnostic Test is an effective diagnostic tool for malaria amongst children in the study population. Primary and secondary health centres in the region should adopt Rapid Diagnostic Test in malaria diagnosis before administration of ACTs to avoid unnecessary treatment.

